# High-Resolution Spatial Distribution and Estimation of Access to Improved Sanitation in Kenya

**DOI:** 10.1371/journal.pone.0158490

**Published:** 2016-07-12

**Authors:** Peng Jia, John D. Anderson, Michael Leitner, Richard Rheingans

**Affiliations:** 1 Faculty of Geo-information Science and Earth Observation (ITC), University of Twente, Enschede, 7500, The Netherlands; 2 Emerging Pathogens Institute, University of Florida, Gainesville, FL, United States of America; 3 Department of Environmental and Global Health, University of Florida, Gainesville, FL, United States of America; 4 Department of Geography and Anthropology, Louisiana State University, Baton Rouge, LA, United States of America; 5 Department of Geoinformatics - Z_GIS, University of Salzburg, Salzburg, Austria; 6 Goodnight Sustainable Development Department, Appalachian State University, Boone, NC, United States of America; Peking UIniversity, CHINA

## Abstract

**Background:**

Access to sanitation facilities is imperative in reducing the risk of multiple adverse health outcomes. A distinct disparity in sanitation exists among different wealth levels in many low-income countries, which may hinder the progress across each of the Millennium Development Goals.

**Methods:**

The surveyed households in 397 clusters from 2008–2009 Kenya Demographic and Health Surveys were divided into five wealth quintiles based on their national asset scores. A series of spatial analysis methods including excess risk, local spatial autocorrelation, and spatial interpolation were applied to observe disparities in coverage of improved sanitation among different wealth categories. The total number of the population with improved sanitation was estimated by interpolating, time-adjusting, and multiplying the surveyed coverage rates by high-resolution population grids. A comparison was then made with the annual estimates from United Nations Population Division and World Health Organization /United Nations Children's Fund Joint Monitoring Program for Water Supply and Sanitation.

**Results:**

The Empirical Bayesian Kriging interpolation produced minimal root mean squared error for all clusters and five quintiles while predicting the raw and spatial coverage rates of improved sanitation. The coverage in southern regions was generally higher than in the north and east, and the coverage in the south decreased from Nairobi in all directions, while Nyanza and North Eastern Province had relatively poor coverage. The general clustering trend of high and low sanitation improvement among surveyed clusters was confirmed after spatial smoothing.

**Conclusions:**

There exists an apparent disparity in sanitation among different wealth categories across Kenya and spatially smoothed coverage rates resulted in a closer estimation of the available statistics than raw coverage rates. Future intervention activities need to be tailored for both different wealth categories and nationally where there are areas of greater needs when resources are limited.

## Introduction

Sanitation facilities refer to latrines and other facilities to contain or safely dispose of human excreta [1, 2]. Access to adequate sanitation facilities is important in reducing the risk of a wide range of adverse health outcomes, including diarrhea, soil-transmitted helminthes infection, schistosomiasis, trachoma, cystocercosis, poor nutritional status, tropical enteropathy, etc. [3–13]. Worldwide, 2.5 billion people still lack access to improved sanitation, and about 1 billion of those practice open defecation [14].

The coverage of improved sanitation is also a key indicator for the socio-economic status of households, which has been repeatedly highlighted by recent efforts [15, 16]. Owing to the significant variance in the economy in many low-income countries, there exists a distinct disparity in sanitation among different wealth groups, which may hinder the progress across each of the Millennium Development Goals (MDGs) [2, 14, 16, 17].

The importance of sanitation facilities with greater quality than quantity has been demonstrated in a range of recent studies in Kenya [18–20]. Jackson [21] indicated that limited financial resources in Kenya are the primary constraint to the large-scale adoption of sanitation technologies more so than a multitude of other reasons (e.g., lack of sanitation and hygiene awareness, knowledge on diseases and how to build latrines). Nevertheless, even the poorest can still afford pit latrines and, in addition, more than half of the households without access to sanitation were willing to invest in facilities and 62% of households with access expressed their willingness to pay for further improvement [21]. Therefore, it is of extreme importance to direct the resources and technologies at the areas of greatest need, and a greater disaggregation and a higher resolution in the resultant datasets are necessary for better decision-making [22].

Location matters in unequal access to improved sanitation in Latin American and Caribbean countries [23]. Some efforts for estimating the urban, rural, and overall population with improved sanitation have been undertaken [14, 22, 24, 25], but few studies were conducted for locating the areas of highest need at higher detail. To fill this gap, the goals of this study are 1) observing the spatial heterogeneity of improved sanitation coverage in different wealth categories and among the overall population at the 100-meter grid cell and county levels, 2) estimating the population covered by improved sanitation based on household survey data and a high-resolution gridded population data product, and 3) comparing the estimates of covered population with the nationwide estimates from the World Health Organization (WHO)/UNICEF Joint Monitoring Program (JMP) for Water Supply and Sanitation [22, 25]. The approach of estimation including time adjustment can be applied to other studies with similar goals.

## Materials and Methods

### Study Area

As one of the countries in East Africa, Kenya is located on the equator and bordered by Tanzania to the south, Uganda to the west, South Sudan to the northwest, Ethiopia to the north, and Somalia to the northeast. It has a land area of approximately 581,082 km^2^ and a population of nearly 40.5 million people according to a 2010 estimate by the World Bank. Following a High Court ruling in September 2009, Kenya is divided into 47 counties that belong to eight provinces (from largest to smallest in area: Rift Valley, Eastern, North Eastern, Coast, Central, Nyanza, Western, and the capital Nairobi). However, the population ranking based on a 2009 Kenya Census is different from the size ranking of the provinces (Table 1). Many Kenyans still live in rural areas (76.8% and 76.4% in 2009 and 2010, respectively), according to the 2011 revision of World Urbanization Prospects (UNPD 2012). The Gross Domestic Product (GDP) per capita (US $) in Kenya during 2009–2013 is $994, higher than the neighbouring countries of Ethiopia ($498), Uganda ($572), and Tanzania ($695), but lower than South Sudan ($1,221) (The World Bank 2014).

**Table 1:** General spatial and demographic information of Kenya.

Province	County Number	Area (km^2^)	Area Rank	Total Population	Pop Rank	Male	Female
Rift Valley	14	183,014	1	10,006,805	1	5,026,462	4,980,343
Eastern	8	153,527	2	5,668,123	2	2,783,347	2,884,776
North Eastern	3	127,355	3	2,310,757	8	1,258,648	1,052,109
Coast	6	82,510	4	3,325,307	6	1,656,679	1,668,628
Central	5	13,168	5	4,383,743	4	2,152,983	2,230,760
Nyanza	6	12,542	6	5,442,711	3	2,617,734	2,824,977
Western	4	8,267	7	4,334,282	5	2,091,375	2,242,907
Nairobi	1	698	8	3,138,369	7	1,605,230	1,533,139
TOTAL	47	581,082		38,610,097		19,192,45	19,417,63

Source: Kenya Census 2009

### Data Collection

Household-level data from the 2008–2009 Kenya Demographic and Health Surveys (KDHS) were collected from a representative sample of 10,000 households grouped into clusters randomly selected within a two-stage sampling design [26, 27]. Each cluster was represented by a GPS point with latitude and longitude coordinates and defined as either an urban or rural cluster [28]. There are 397 total clusters included in this study after initial data quality control and processing, with 384 clusters including 19–25 households and the remaining 13 clusters including 9–18 households. Among all the surveyed clusters, 132 are urban and 265 are rural clusters. Their actual locations were offset towards a random direction by a maximum of 2 kilometers for urban clusters and a maximum of 5 kilometers for rural clusters, with a further 1% of the rural clusters displaced a maximum of 10 kilometers, to maintain confidentiality of the surveyed respondents.

**Fig 1:**
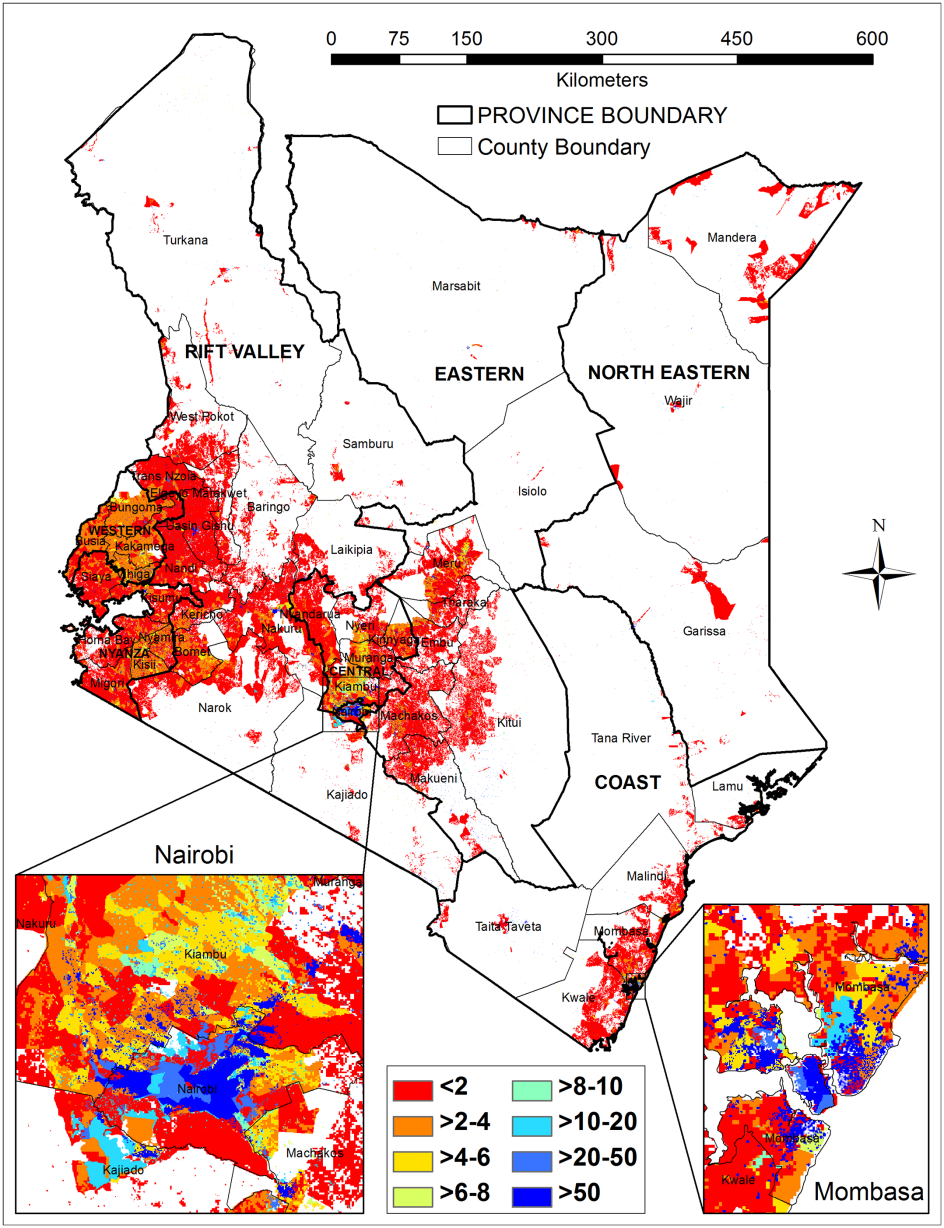
Spatial distribution of the population in Kenya (Unit: persons/ha).

The *AfriPop* project, now becoming part of the *WorldPop* project, is at present the best freely available source of data for population-related studies in Africa and in most cases it has outperformed the *African Population Database* (APD), *Global Rural-Urban Mapping Project* (GRUMP), *Gridded Population of the World version 3* (GPW3), and *LandScan* in Kenya [29]. The *AfriPop 2010*, adjusted to match UN national estimates with a resolution of 100 meters, was used in this study to represent the population density (persons/ha) (Fig 1).

### Data Processing

Drinking water supply and sanitation facilities have been included as household assets in the default asset scores and quintiles of DHS datasets [17]. Therefore, an asset score for each household was constructed using Principal Components Analysis (PCA) in *Stata 12* [30], on the basis of household ownership of durable goods and housing characteristics excluding sanitation and drinking water supply [31, 32]. According to the asset scores, all households in Kenya were ranked and divided into five ordered subgroups called wealth quintiles, where the first quintile included the poorest 20% households and the fifth quintile included the wealthiest 20%.

Five subsets of clusters representing five quintiles were created, each of which included all of the clusters that contained at least one household in the corresponding quintile. It is worth noting that those five subsets were not mutually exclusive. As the surveyed households in one cluster might fall in multiple quintiles but be only represented by one GPS point, a cluster can appear in all subsets only if it included at least one household in the corresponding wealth quintile.

An improved sanitation facility was defined by JMP as one that ‘hygienically separates human excreta from human contact’, for MDG monitoring [14]. In this study, five types of toilet facilities are defined as improved: (1) Pit latrines with slabs, (2) ventilated improved pit latrines (VIP), (3–5) toilets that flush or pour to piped sewer systems/septic tanks/pit latrines. Pit latrines without slabs and flush toilets that do not have an outflow to an appropriate site were considered unimproved sanitation. Shared and private facilities were included provided they met the definition of improved facilities. The percentage of households with any of the five improved types in each cluster was calculated as the coverage rate of improved sanitation for the overall population and for each quintile and accounted for the survey design and weights in *Stata 12* [30].

### Data Analysis

Excess risk in the overall population and in each wealth quintile was assessed where the risk for lack of improved sanitation was higher (excess ratio risk >1) and lower (excess ratio risk <1) than the national average level, and where extremely high risk was present (excess ratio risk >2). The national average level was calculated as the ratio between the total number of households without improved sanitation and the number of all surveyed households, instead of simply using the average of all clusters. The excess risk in each quintile was calculated as:

$$r_{cq}=(n_{cq}/N_{cq})/\left(\underset{\text{x}\in \text{P}}{\sum }n_{x}/\underset{\text{x}\in \text{P}}{\sum }N_{x}\right)$$

where *r*_cq_ = excess risk for the cluster c in quintile q, *n*_cq_ = number of households without improved sanitation in cluster c in quintile q, *N*_cq_ = total number of households in cluster c in quintile q, *n*_x_ = total number of households without improved sanitation in cluster x, *N*_x_ = total number of surveyed households in cluster x, and P represents all 397 clusters in this study.

A Thiessen polygon was created around each cluster, within which any location was closer to the centered cluster than other surrounding clusters. According to Tobler’s first law of geography that ‘everything is related to everything else, but near things are more related than distant things [33],’ spatial smoothing was used to produce a corresponding estimate to the raw coverage rate of each cluster from a collection of neighboring clusters enclosed by Thiessen polygons. The first order rook contiguity was applied as the spatial smoothing rule in this study, meaning that all neighboring polygons sharing a border of some length that is longer than a point-length border, with the target Thiessen polygon were defined as neighbors.

Local Moran’s I were calculated using *GeoDa* [34] to identify clusters of high and low coverage of improved sanitation, where a Random Permutation Procedure (RPP) was additionally used to replicate the statistics 999 times to generate reference distributions. By comparison with reference distributions, the statistical significance of high and low spatial clustering was tested. The Local Moran’s I was calculated for both raw and spatially-smoothed rates.

### Interpolation

There are two broad classes of interpolation methods, deterministic (for example, inverse distance weighted (IDW) interpolation) and probabilistic (for example, Kriging interpolation), both of which were used in this study to predict values where sampling was not conducted [35]. According to Tobler’s first law of geography, the IDW interpolation uses the measured values surrounding each unsampled location, weighted by the proximity to this location, to predict the value.

A semivariogram is a function of the distance and direction separating two locations, which is estimated from the data for quantifying the spatial dependence in the data and assumed to be the same at all locations (spatial homogeneity) in the classical Kriging method. This assumption rarely holds true in practice, so the empirical Bayesian Kriging (EBK) interpolation was used to account for the variance by estimating multiple semivariogram models from the data instead of a standalone semivariogram. The selection of models and parameters in the common Kriging methods, such as ordinary and universal Kriging, was automated in the EBK.

The raw and spatially smoothed coverage rates of improved sanitation in the overall population and in each quintile were interpolated by the IDW and EBK based on all clusters that included at least one household in that quintile. The root mean squared error (RMSE) was calculated by omitting a measured value then predicting its value based on surrounding point values. The measured and predicted values were compared through an iterative cross validation process within each model to compare the level of accuracy different models predicted as values at unobserved sites. The IDW and EBK interpolation models for the overall population and for each quintile were both adjusted to obtain a minimal RMSE, and the ones with the smaller RMSE, either IDW or EBK, were selected as final models.

### Estimating the Population with Improved Sanitation

Both the continuous raw and spatially-smoothed coverage rates of improved sanitation for the overall population, interpolated by final models, were re-sampled to spatially match the actual population distribution in *AfriPop*, and also adjusted separately in urban and rural areas for temporally matching the 2010 *AfriPop*. Adjustment rates between 2008–2010 for urban and rural areas were determined by linear regression, established based on multiple surveys during 1989–2008, including DHS (1989, 1993, 1998, 2003 and 2008), Census (1989 and 1999), Welfare Monitoring Survey (1994), Core Welfare Monitoring Questionnaire (1997), Multiple Indicator Cluster Survey (2000), World Health Survey (2004), and National Bureau of Statistics (2006) [36].

Due to a lack of city boundaries, the urban areas were extracted under the assumption that the population density is greater in the city than outside. According to the World Bank estimates, 24% of the total population in Kenya lived in urban areas in 2010 (23% in 2009, and also 24% in 2011 and 2012). All grid cells were ranked in order from highest to lowest population density. Starting with the highest population density, all cells including 24% of the total population were extracted as urban areas in this study, and the remaining areas were marked as rural. The total number of the population covered by improved sanitation was the sum of urban and rural estimates, separately calculated by adding together all grid cell values by time-adjusted coverage rates and population.

Additionally, the interpolated raw and spatially-smoothed coverage rates of improved sanitation for the overall population and each quintile were masked with the populated areas (population density ≥ 1 person/km^2^, namely ≥ 0.01 person/ha in the *AfriPop 2010*), and then averaged at the county level for a better view.

## Results

### Raw Coverage

**Fig 2:**
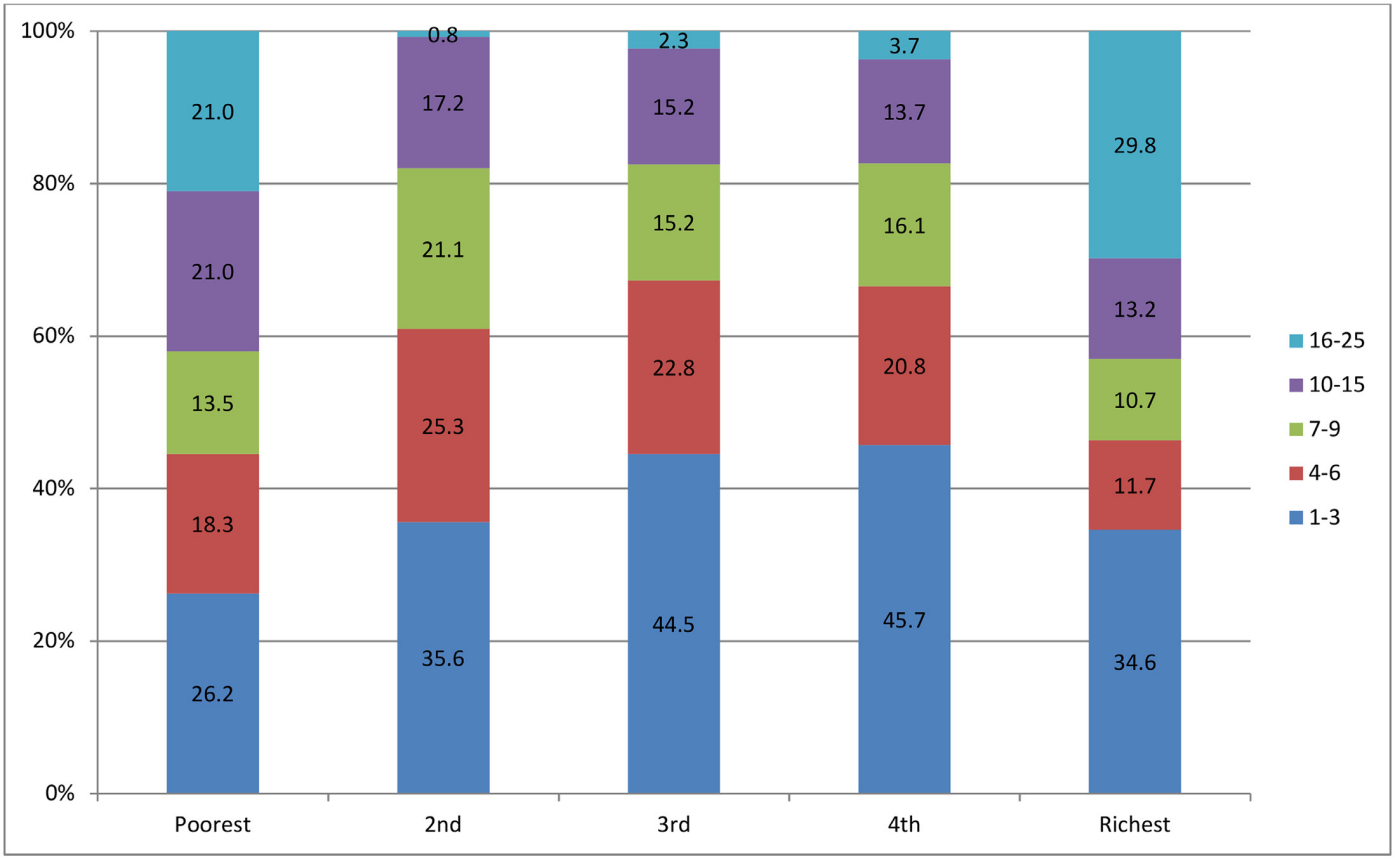
Numbers and percentages of clusters categorized by the number of households each cluster represents, presented for all clusters by quintile.

**Fig 3:**
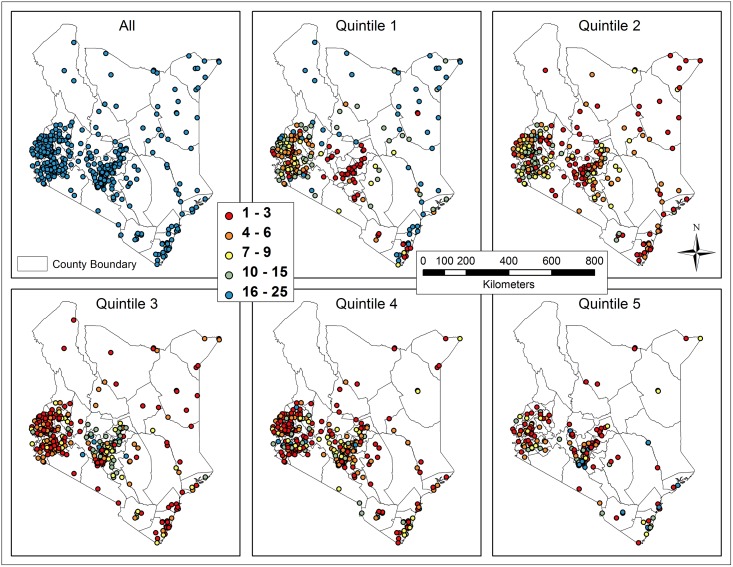
Number of households in each cluster for the overall population and each subset (quintile).

Each household was assigned to one of five quintiles, according to its national asset scores. The numbers and percentages of clusters with different households in each subset (quintile) are shown in Fig 2. When comparing the distribution of surveyed households for the five quintiles (Fig 3), we found a clear transition of wealth from periphery to the capital Nairobi. Most of the households in quintile 1 are located in the north and west (Western and Nyanza Province) of Kenya and southeastern coastline regions (Coast Province). The households in quintile 2 were primarily clustered in the west, with the clustering trend in the north and southeastern coastlines waning. In quintile 3, an obvious clustering trend emerged in the Central Province and this trend continued into quintile 4. The majority of the richest 20% households in quintile 5 were congregated in Nairobi to the south of the Central Province, and in Mombasa in the southeast, the second largest city in Kenya.

**Fig 4:**
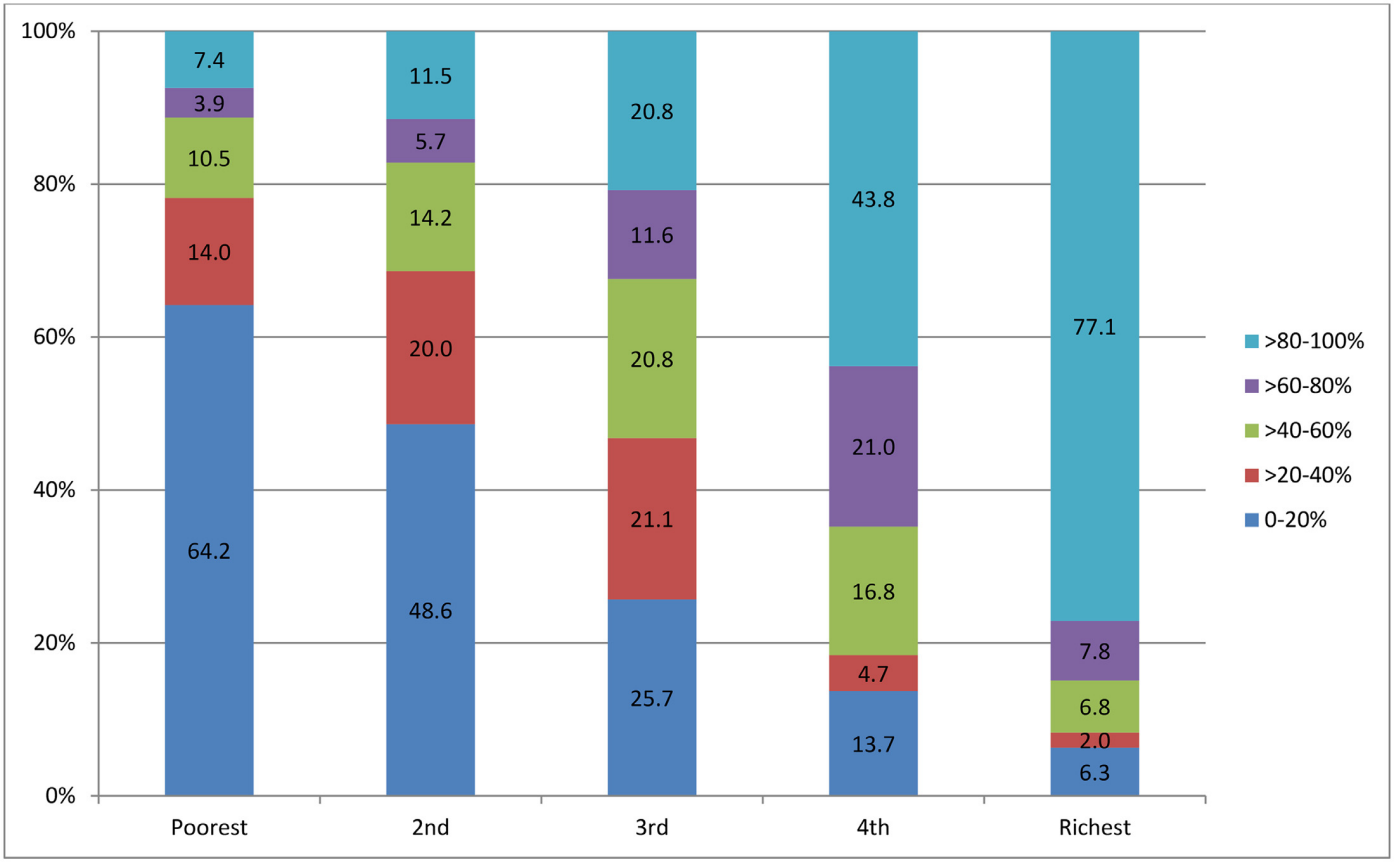
Numbers and percentages of clusters categorized by the raw coverage rates of improved sanitation, presented for all clusters by quintile.

The numbers and percentages of sampled clusters with varying coverage of improved sanitation in each quintile are shown in Fig 4, where a clear rise and decline in percentage of the clusters with 80–100% and 0–20% coverage, respectively, were indicated from quintile 1 to 5. The differences in raw coverage rates among the sampled clusters between each subsequent and preceding quintile were statistically tested (Table 2). It was obvious that the coverage rates of improved sanitation have continuously increased from the poorest to the richest quintile, with significant differences between each subsequent and preceding quintile (α ≤ 0.001).

**Table 2:** Results of 2-tailed *t*-tests for the differences in the coverage rates of improved sanitation between each subsequent and preceding quintile.

	Levene’s Test		*t*-test for Equality of Means						
Quintile	F.	Sig.	t	df	Sig.	Mean Difference	Std. Error Difference	95% Lower	95% Upper
2 versus 1	3.623	0.058	-3.292	488	0.001	-0.092	0.028	-0.146	-0.037
3 versus 2	3.724	0.054	-5.566	562	0.000	-0.157	0.028	-0.213	-0.102
4 versus 3	1.549	0.214	-7.866	623	0.000	-0.214	0.027	-0.267	-0.160
5 versus 4	22.702	0.000	-6.342	525	0.000	-0.176	0.028	-0.230	-0.121

*Equal variances assumed

**Fig 5:**
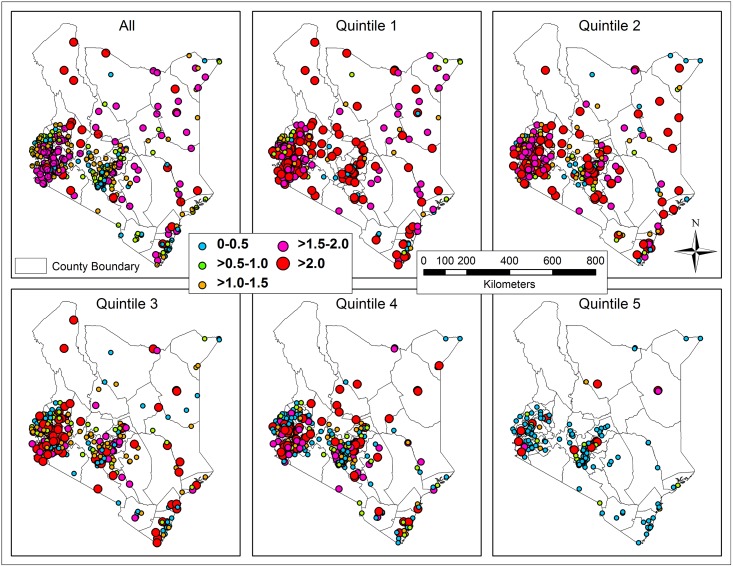
Excess risk for sanitation-related diseases in clusters for the overall population and each subset (quintile).

Excess risk was mapped overall and for each quintile to illustrate the degree of potential risks for sanitation-related diseases due to low coverage (Fig 5). The clusters with highest risks relative to the national average were highlighted with larger rose and red points. The distribution of high risks was generally consistent with that of low coverage and clusters having households in the poorest quintiles. For example, quintile 1 has the highest number of red points representing clusters with extremely high excess risk (ranging from 2 to 11.4). In contrast, clusters with households in quintile 5 showed lower excess risk, with most clusters falling into the low excess risk range (0–0.5).

**Fig 6:**
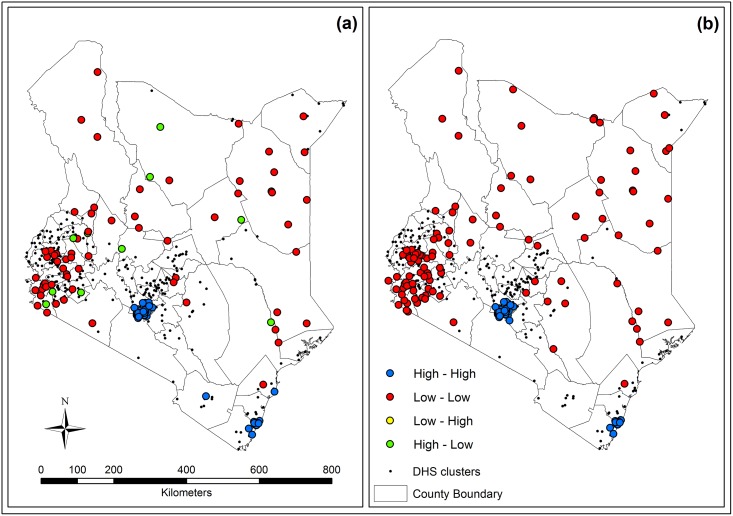
Clustering trends of coverage rates in cluster before (a) and after spatial smoothing (b).

Local Moran’s I was calculated for the overall population (Fig 6a), demonstrating the clustering of high (High-High) and low coverage (Low-Low), high surrounded by low coverage (High-Low), and low surrounded by high coverage (Low-High). The majority of clusters with high coverage were located in Nairobi, Kiambu County (Central Province) that bordered Nairobi to the north, and Mombasa. Some high coverage clusters were sparsely spread across neighboring counties around Mombasa, including Malindi County (Coast Province) to the north of Mombasa, Kwale County (Coast Province) to the south of Mombasa, and Taita Taveta County (Coast Province) to the northwest of Mombasa. Most clusters in northern and western Kenya had lower coverage, especially in the south of the Western Province and around the branch of Lake Victoria that stretches to Nyanza Province in the southwest, called Lake Victoria region hereafter. Although there were few clusters with high coverage in the north and west of Kenya, each of these clusters was surrounded by clusters with poor coverage. Clustering with low coverage was found in the Lake Victoria region and northern rural regions.

**Fig 7:**
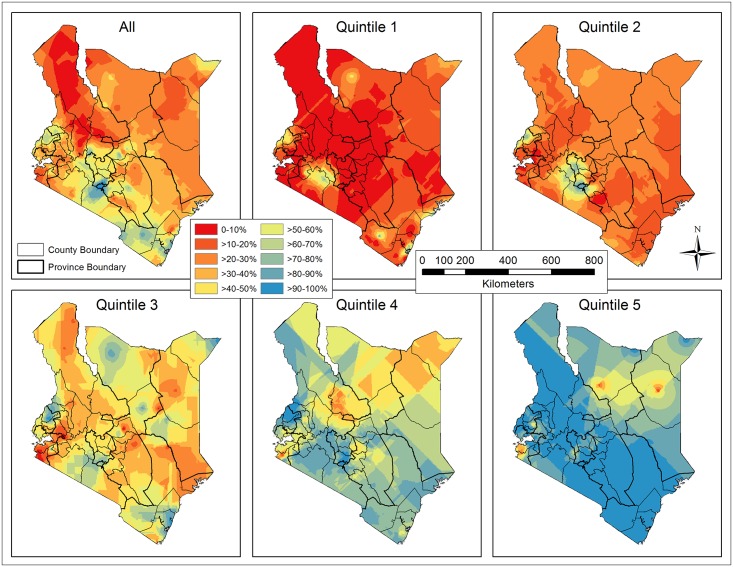
Kenya map of the raw coverage rates of improved sanitation interpolated by Empirical Bayesian Kriging (EBK) for the overall population and each subset (quintile).

**Fig 8:**
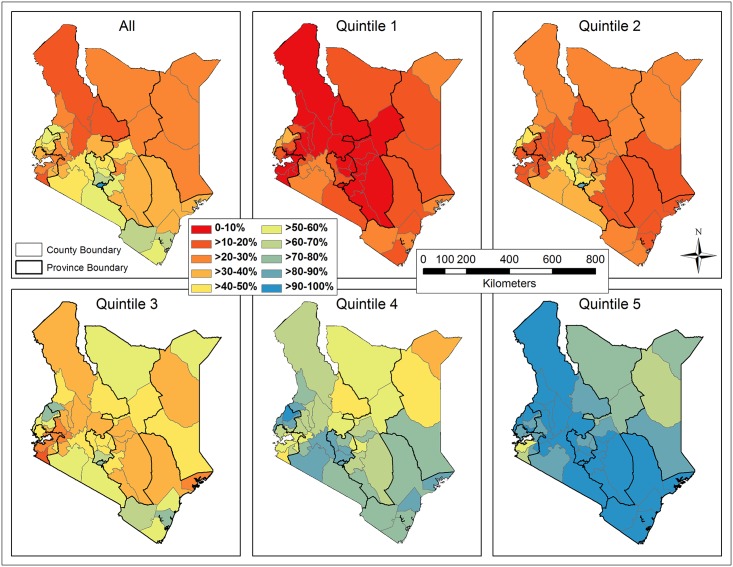
Raw coverage rates of improved sanitation averaged by county for the overall population and each subset (quintile).

The raw coverage rates of improved sanitation were interpolated based on surveyed households by EBK interpolation (Fig 7), which produced less RMSEs than IDW for the overall population and for the five quintiles (Table 3). The general trend in coverage rates of improved sanitation has increased from quintile 1 to 5, due primarily to the elevated rates at sampled clusters. A balance for the resolution of spatial heterogeneity was found, where interpolated coverage rates were re-sampled, masked with the populated areas (≥ 0.01 person/ha), and averaged by county (Fig 8). Except Bungoma (58.7%) and Trans Nzoia County (53.0%) in the west, nine out of eleven counties with more than 50% coverage rates were located in the south, forming a continuous band from Nyandarua (54.9%) to Kwale (52.7%), going through Muranga (52.1%), Kiambu (66.0%), Nairobi (94.0%), Machakos (52.1%), Kajiado (59.9%), Taita Taveta (63.2%), and Mombasa (60.5%). The coverage rates in the south, decreasing from Nairobi in all directions, were generally higher than in the north and east, while Nyanza and North Eastern Province had relatively poor coverage.

**Table 3:** Numbers and percentages of clusters categorized by smoothed improved sanitation coverage rates, presented for all clusters by quintile.

	Total	Raw		Spatial	
Quintile	number	EBK	IDW	EBK	IDW
1	229	0.265	0.286	0.059	0.065
2	261	0.286	0.305	0.074	0.076
3	303	0.311	0.332	0.087	0.091
4	322	0.303	0.314	0.084	0.089
5	205	0.226	0.242	0.062	0.066
ALL	397	0.215	0.234	0.064	0.070

### Spatially-Smoothed Coverage

**Fig 9:**
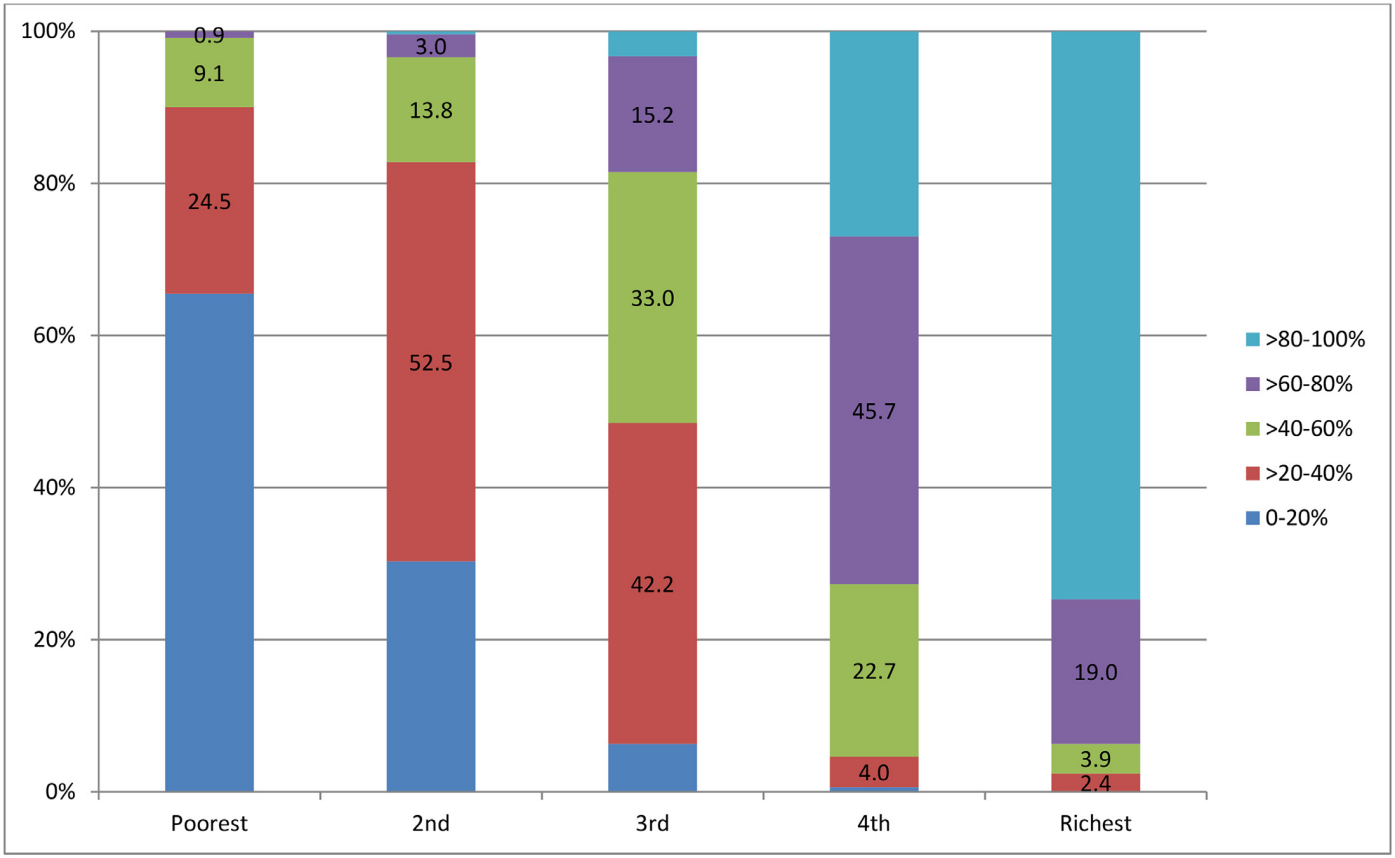
Numbers and percentages of clusters categorized by the spatially-smoothed coverage rates of improved sanitation, presented for all clusters by quintile.

Similarly, a spatially-smoothed coverage rate was calculated for each cluster from a collection of its neighboring clusters to overcome the small number issue that may happen in this study, since 26.2%-45.7% of clusters in separate quintile subsets included only three households or less (Fig 2). The spatially-smoothed coverage rates had stronger correlation with the predicted coverage rates, which were produced by omitting a point and calculating the rate of this location based on the remaining points. The numbers and percentages of clusters with different smoothed coverage in each quintile are shown in Fig 9. The percentages of clusters with high coverage in quintile 1 and 2, and of the clusters with low coverage in quintile 4 and 5 declined significantly, which resulted from spatial smoothing that adjusted the raw coverage rates of clusters in each quintile towards a level suitable for the average economic status at that level. Quintile 3 was a middle-level status, so the raw coverage rates in clusters at two ends were adjusted towards the middle with the percentages of the clusters with 0–20% and >80–100% coverage dwindling and of the clusters with >20–80% coverage growing.

**Fig 10:**
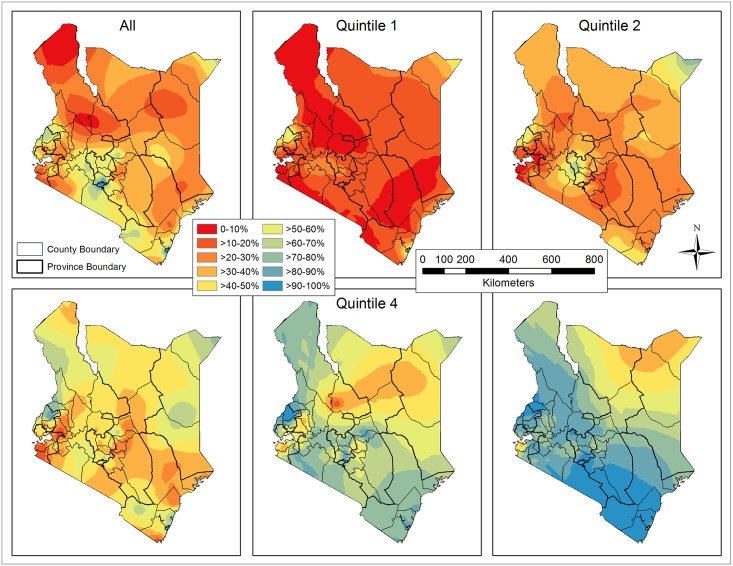
Kenya map of the spatially-smoothed coverage rates of improved sanitation interpolated by Empirical Bayesian Kriging (EBK) for the overall population and each subset (quintile).

**Fig 11:**
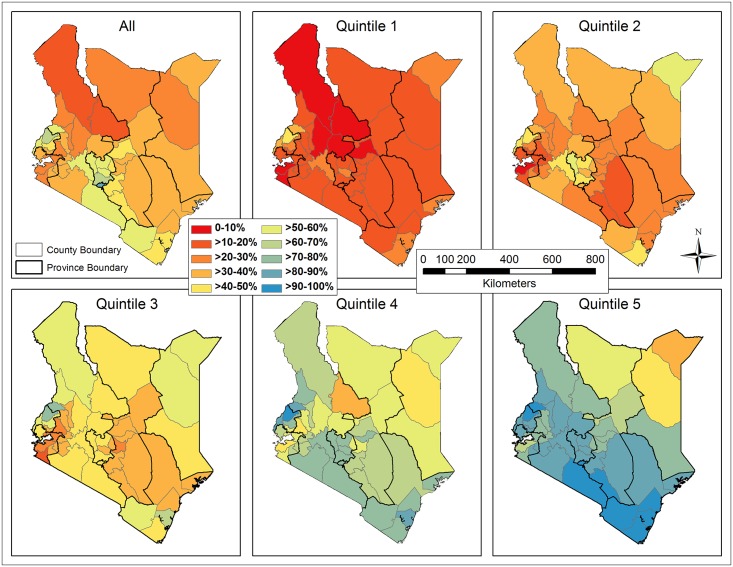
Spatially-smoothed coverage rates of improved sanitation averaged by county for the overall population and each subset (quintile).

**Fig 12:**
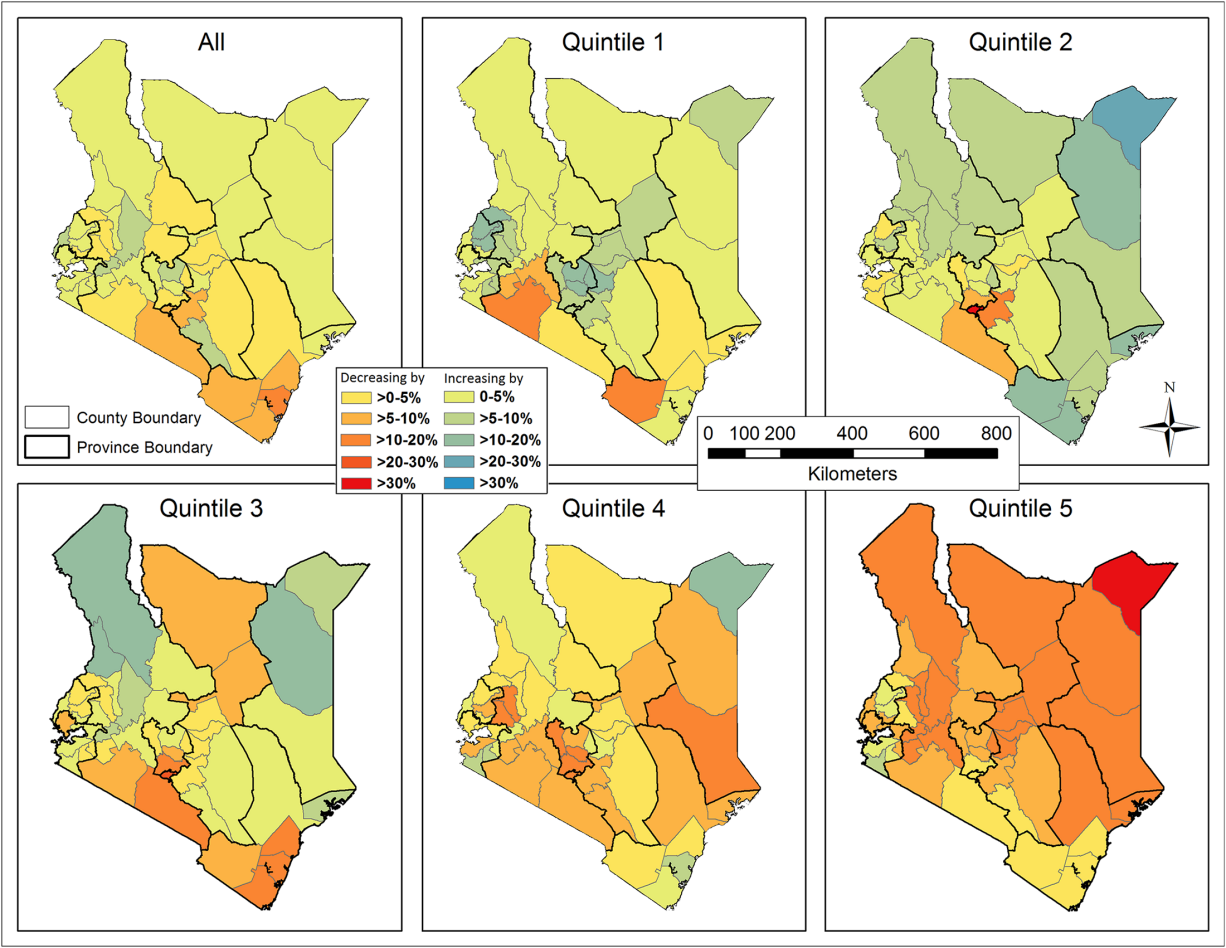
Differences between the raw and spatially-smoothed coverage rates of improved sanitation for the overall population and each subset (quintile) on the county level.

The spatially-smoothed coverage rates interpolated on the basis of sampled clusters were shown in Fig 10, where EBK remained superior to IDW. Likewise, the continuous smoothed rates were re-sampled, masked with populated areas (population density ≥ 1 person/km^2^), and averaged by county (Fig 11). The direction (increase or decrease) and degree of spatial smoothing in all counties, measured by subtracting the raw from spatially-smoothed coverage rates, were categorized (Fig 12). In general, the spatially-smoothed coverage rates decreased in the south (surrounding Nairobi), and increased in the north and west (surrounding Lake Victoria), with the degree of being spatially smoothed varying by wealth and across Kenya. In the subsets, the rates in quintile 1 and 5 were most intensively under- and over-estimated, respectively. The degree of spatial smoothing in each quintile was examined against the numbers of surveyed households within clusters. A consistent trend in all five quintiles indicated that the coverage in clusters including fewer households was more drastically changed by spatial smoothing.

Local Moran’s I was also calculated for the overall population after spatial smoothing (Fig 6b). The general clustering trend of hot and cold spots among sampled clusters was confirmed after smoothing, although clustering of spatial outliers was eliminated.

### Estimated Population with Improved Sanitation

Among the surveyed clusters by the 2008–2009 KDHS, mean coverage rates were 82.1% and 35.1% in urban and rural regions, respectively, while the median rates were 91.5% and 20.8% in urban and rural areas, respectively. According to the linear regression, the estimated urban coverage rates in 2008 and 2010 were 78% and 79.2%, respectively, while the estimated rural coverage rates in 2008 and 2010 were 50% and 50.5%, respectively. Therefore, growth rates of 1.2% and 0.5% were added to the interpolated coverage rates in urban and rural grid cells, respectively, under the assumption that the coverage rate has increased by the same extent nationwide during 2008–2010.

Adding together all products by time-adjusted coverage rates and total population, the estimated total population with improved sanitation in 2010, based on raw and spatially-smoothed rates, were 19,875,164 (6,569,164 in urban and 13,306,000 in rural regions) and 18,978,948 (6,084,058 in urban and 12,894,890 in rural areas), respectively. When divided by the total population, the estimated overall coverage rates, before and after spatial smoothing, were 49.1% (67% in urban and 43.5% in rural regions) and 46.9% (62.1% in urban and 42% in rural areas), respectively.

According to annual updates of the Progress on Drinking Water and Sanitation, nationwide estimates of coverage rates of improved sanitation (improved + shared) in 2008 were 78% and 50% for urban and rural Kenya, respectively [22]. According to the 2012 update report, estimates for urban and rural Kenya in 2010 were 80% and 53%, respectively [25]. However, estimates for urban areas in Kenya in 2011 [14] and 2012 [24] were only 78% and 79%, respectively, while estimates for rural Kenya were 48% in both 2011 and 2012. It implies that the coverage rates in 2010 either peaked in that year or was overestimated in 2012. Also considering estimates in the preceding (2008) and subsequent year (2011), a reasonable range of estimates in 2010 by JMP should be about 78–80% in urban areas and 48–50% in rural areas. As a result, our estimation based on the coverage rates of improved sanitation before spatial smoothing provides a close approximation of the JMP estimates.

## Discussion

Declared as the International Year of Sanitation, the year 2008 serves as an important baseline for assessing the progress towards the Millennium Development Goal (MDG) target to reduce by half the proportion of the 2.6 billion people without access to basic sanitation by 2015. Therefore, a basic understanding of the status quo of the sanitation in the baseline year is a necessary requirement for future strategies. In addition to several recent and encouraging studies in Kenya, the availability of data sources in proximate years (improved sanitation coverage rates in 2008 and population in 2010) offers the possibility to develop a spatial approach for estimating the size and location of the population covered by improved sanitation.

This is the first study to comprehensively analyze the spatial distribution of improved sanitation coverage among Kenyan households by their socioeconomic status at the county and even finer levels. Among all surveyed households, we found that wealthier populations were located in central regions in or near Nairobi and poorer populations were dispersed outwards from Nairobi in all directions, particularly in the northwest and northeast, and Lake Victoria region. The patterns of high coverage of improved sanitation were generally consistent with locations where wealthier populations lived. Wealthier populations were often collocated in areas with the most improved sanitation services and poorer populations were often collocated in areas with less effective sanitation services.

Excess risk and Local Moran’s I showed the relationship between sanitation nationally and across socioeconomic status using different parameters. The former showed the extent of potential risk in surveyed clusters relative to the national average of households without sanitation, independent of surrounding clusters. The latter showed the relationship of improved sanitation coverage for each cluster to neighboring clusters. Excess risk in each quintile indicated that a gap of coverage existed among households with comparable socioeconomic status. Clusters with the poorest households (quintile 1) had more than twice the excess risk relative to the national average in both clusters around urban areas of Nairobi and Kisumu as well as in urban areas in northern Kenya. Households in quintile 3 generally had lower risk for lack of improved sanitation than the poorest households, but still show significant excess risk, especially around urban areas. Significantly higher risks appeared in the west and middle regions in quintile 4, possibly as the clusters had greater wealth heterogeneity, with richer households located near middle and poorer households. Additionally, a minor clustering of low coverage in quintile 4 occurred in the Lake Victoria region.

Most clusters fell into two categories, namely hot spots and cold spots, indicating that areas of high and low coverage are often surrounded by neighboring clusters with similar coverage rates of improved sanitation. This is important in deciding areas of highest priority for risk for sanitation-related diseases. In contrast, high coverage clusters were infrequently surrounded by low coverage clusters and such a relationship was dispersed across Kenya. Local Moran’s I using spatial smoothing coverage likewise considered not only the coverage in each cluster but also in their neighborhoods, to adjust outliers to a local average level. Most of the noticeably adjusted clusters had inconsistent raw rates within neighborhoods or small numbers of households in quintile subsets. For example, rates in most clusters with less than three households in all quintiles were adjusted, but in most clusters with more than ten households the rates remained or were only slightly adjusted. In addition to adjusting possible outliers, spatial smoothing also more clearly showed the same trend resulting from raw coverage estimates. Further surveys and analytical methods are needed to assess the accuracy of spatial smoothing used to adjust inconsistent rates, especially in regions with large differences in coverage before and after smoothing.

The IDW interpolation, widely used in many socioeconomic, disease, and health studies [37–40], was used for comparison purposes. Since the numbers of households in clusters varied considerably in quintile subsets, from 1 to 25 depending on the national asset scores of households, we used the numbers of households to weight the clusters for the IDW interpolation under the assumption that the rates in clusters with more surveyed households were more reliable. However, the RMSE from the IDW was higher compared to the EBK interpolation in all five subsets and the overall dataset, which demonstrated the strengths of the EBK for small datasets over not only other kriging methods, but also the deterministic interpolation approach.

A primary concern was the representativeness of surveyed clusters. The low coverage was partly attributed to rural clusters across Kenya. Fewer clusters were sampled in the northern and eastern regions than in the central and western regions. Spatial patterns of coverage in wealth quintiles were in part attributed to the sampling strategy. For instance, most of the surveyed households in the north were located in rural areas with very low population density, while all surveyed households in Nairobi and Mombasa were in urban areas. Furthermore, all households in each of 397 clusters were sparsely sampled instead of completely surveyed, subjecting them to some level of bias.

There is an ongoing debate about whether shared facilities are able to provide a similar level of protection as private ones [17]. In this study, we included the households with all types of improved sanitation facilities regardless of their status as shared or private. When averaging the coverage rates by county, all populated areas within each county were given the same weight under the assumption that the population density was homogeneous across each county. Additional approaches should be explored to seek more fairly representative values of the average level in each county. Additionally, spatial smoothing may misinterpret some truly abnormal values as outliers, which would introduce new sources of error and mitigate the degree of true spatial heterogeneity.

The estimates from our approach, even prior to spatial smoothing, are apparently lower than the JMP estimates in both urban and rural areas, most likely due to the random shift of the original location of the surveyed urban clusters away from the actual urban areas. This resulted in the urban clusters not matching the underlying urban grid cells in *AfriPop*. Therefore, when calculating the total urban population with improved sanitation, the coverage rate was most likely multiplied by the underlying rural grid cells with relatively low population density, instead of the correct urban grid cells with high population density. This greatly reduced the size of covered population in urban areas. In contrast to urban regions, rural regions possess relatively large areas, which made estimates of the rural coverage rates less subjective to the mismatch resulting from the random shift in the original data. Another limitation of our estimation approach may lie in the assumption that the population density is greater in the city than outside the city when determining urban boundaries. Some rural areas may be misclassified as urban areas due to their high population density. However, using an only cut-off value of population density to extract urban boundaries is considered more arbitrary by comparison, as the cut-off value may vary over time and across countries. We thus took advantage of the strengths of the high-resolution *AfriPop* population grids, which captured the clustering of the population at a finer resolution.

This study suggests that interpolating and time-adjusting coverage rates in sampled clusters in DHS, matching with and multiplying by *AfriPop* population grids, is a feasible and acceptable approach to estimate the size of the population with improved sanitation and their spatial distribution, especially for the rural areas. Due to the limited urban areas in low- and middle-income countries, the estimates in the urban areas need to be explained with caution. This study adds to the growing body of literature highlighting the importance of improved sanitation, demonstrating spatial evidence for future intervention activities to be tailored for both different wealth categories and nationally where there are areas of greater needs when resources are limited.

## References

[1] Clasen T, Schmidt W-P, Rabie T, Roberts I, Cairncross S. Interventions to improve water quality for preventing diarrhoea: systematic review and meta-analysis. British Medical Journal. 2007;334(7597):782–5. 10.1136/bmj.39118.489931.BE 000246037300028. 17353208 PMC1851994

[2] Mara D, Lane J, Scott B, Trouba D. Sanitation and health. PLoS medicine. 2010;7(11):e1000363 10.1371/journal.pmed.1000363 21125018 PMC2981586

[3] Andrade IG, Queiroz JW, Cabral AP, Lieberman JA, Jeronimo SMB. Improved sanitation and income are associated with decreased rates of hospitalization for diarrhoea in Brazilian infants. Transactions of the Royal Society of Tropical Medicine and Hygiene. 2009;103(5):506–11. 10.1016/j.trstmh.2008.12.017 19215948

[4] Barreto ML, Genser B, Strina A, Teixeira MG, Assis AMO, Rego RF, et al Impact of a citywide sanitation program in Northeast Brazil on intestinal parasites infection in young children. Environmental health perspectives. 2010;118(11):1637–42. 10.1289/ehp.1002058 20705544 PMC2974706

[5] Black RE, Cousens S, Johnson HL, Lawn JE, Rudan I, Bassani DG, et al Global, regional, and national causes of child mortality in 2008: a systematic analysis. Lancet. 2010;375(9730):1969–87. 10.1016/S0140-6736(10)60549-1 . 20466419

[6] Daniels DL, Cousens SN, Makoae LN, Feachem RG. A case-control study of the impact of improved sanitation on diarrhoea morbidity in Lesotho. Bulletin of the World Health Organization. 1990;68(4):455–63. 2208559 PMC2393155

[7] Esrey SA, Potash JB, Roberts L, Shiff C. Effects of improved water supply and sanitation on ascariasis, diarrhoea, dracunculiasis, hookworm infection, schistosomiasis, and trachoma. Bulletin of the World Health Organization. 1991;69(5):609 1835675 PMC2393264

[8] Humphrey JH. Child undernutrition, tropical enteropathy, toilets, and handwashing. Lancet. 2009;374(9694):1032–5. 10.1016/S0140-6736(09)60950-8 . 19766883

[9] Kotloff KL, Nataro JP, Blackwelder WC, Nasrin D, Farag TH, Panchalingam S, et al Burden and aetiology of diarrhoeal disease in infants and young children in developing countries (the Global Enteric Multicenter Study, GEMS): a prospective, case-control study. Lancet. 2013;382(9888):209–22. 10.1016/S0140-6736(13)60844-2 . 23680352

[10] Lee L, Rosenzweig MR, Pitt MM. The effects of improved nutrition, sanitation, and water quality on child health in high-mortality populations. Journal of Econometrics. 1997. 6873056970129114281related:qYSROeYAYl8J.

[11] Norman G, Pedley S, Takkouche B. Effects of sewerage on diarrhoea and enteric infections: a systematic review and meta-analysis. Lancet Infectious Diseases. 2010;10(8):536–44. 10.1016/S1473-3099(10)70123-7 000280887300013. 20620115

[12] Tate JE, Burton AH, Boschi-Pinto C, Steele AD, Duque J, Parashar UD, et al 2008 estimate of worldwide rotavirus-associated mortality in children younger than 5 years before the introduction of universal rotavirus vaccination programmes: a systematic review and meta-analysis. The Lancet Infectious Diseases. 2012;12(2):136–41. 10.1016/S1473-3099(11)70253-5 16174309925369854786related:Qru-lC6xduAJ. 22030330

[13] Ziegelbauer K, Speich B, Mäusezahl D, Bos R, Keiser J, Utzinger J. Effect of Sanitation on Soil-Transmitted Helminth Infection: Systematic Review and Meta-Analysis. PLoS medicine. 2012;9(1):e1001162 10.1371/journal.pmed.1001162 15949875024031966709related:9eVMC9RWWd0J. 22291577 PMC3265535

[14] WHO UNICEF. Progress on Sanitation and Drinking-Water: 2013 Update. WHO/UNICEF Joint Monitoring Programme for Water Supply and Sanitation (JMP), 2013 6 14. Report No.: 978 92 4 150539 0.

[15] Watkins K. Human Development Report 2006—Beyond Scarcity: Power, poverty and the global water crisis. United Nations Development Programme (UNDP). 2009 8922197817941590799related:D4MswqQD0nsJ.

[16] UNICEF. Progress for Children: Achieving MDGs with Equity. New York, NY: UNICEF, 2010 9 01. Report No.: 978-92-806-4537-8.

[17] Rheingans R, Cumming O, Anderson J, Showalter J. Estimating inequities in sanitation-related disease burden and estimating the potential impacts of pro-poor targeting. London: SHARE: Sanitation and Hygiene Applied Research for Equity, 2012 6 03. Report No.

[18] Dreibelbis R, Greene LE, Freeman MC, Saboori S, Chase RP, Rheingans R. Water, sanitation, and primary school attendance: a multi-level assessment of determinants of household-reported absence in kenya. International Journal of Educational Development. 2013;33(5):457–65.

[19] Greene LE, Freeman MC, Akoko D, Saboori S, Moe C, Rheingans R. Impact of a school-based hygiene promotion and sanitation intervention on pupil hand contamination in Western Kenya: a cluster randomized trial. Am J Trop Med Hyg. 2012;87(3):385–93. Epub 2012/07/18. 10.4269/ajtmh.2012.11-0633 22802437 PMC3435337

[20] Freeman MC, Snel M, Yousif MEF, Gitahi S, Khan F, Wachira S, et al The usage of urinals in Kenyan schools. Waterlines. 2012;31(3):226–39.

[21] Jackson B. Sanitation and Hygiene in Kenya: Lessons on What Drives Demand for Improved Sanitation. The Water and Sanitation Program, 2004 7 13. Report No.

[22] UNICEF, WHO. Progress on Sanitation and Drinking Water: 2010 Update. 2010.

[23] de Barros RP. Measuring inequality of opportunities in Latin America and the Caribbean: World Bank Publications; 2009.

[24] UNICEF, WHO. Progress on Drinking Water and Sanitation: 2014 Update. 2014.

[25] WHO. Progress on Drinking Water and Sanitation: 2012 Update. 2012.

[26] ICF International. Demographic and Health Survey Sampling and Household Listing Manual. Calverton, MD: MEASURE DHS, 2012 10 10. Report No.

[27] Kenya National Bureau of Statistics (KNBS), ICF Macro. Kenya Demographic and Health Survey 2008–09. Calverton, MD: KNBS and ICF Macro: 2010.

[28] ICF Macro. Incorporating Geographic Information Into Demographic and Health Surveys: A Field Guide to GPS Data Collection. Calverton, MD: ICF Macro, 2011 8 24. Report No.

[29] Tatem AJ, Noor AM, von Hagen C, Di Gregorio A. High Resolution Population Maps for Low Income Nations: Combining Land Cover and Census in East Africa. PLOS ONE. 2007. 15945586518798858480related:8HhDnHQaSt0J. 10.1371/journal.pone.0001298 PMC2110897 18074022

[30] StataCorp. Stata Statistical Software: Release 12. College Station, TX: StataCorp LP; 2011.

[31] Filmer D, Pritcett LH. Estimating Wealth Effects Without Expenditure Data—or Tears: An Application to Educational Enrollments in States of India. Demography. 2001;38(1):115–32. 7735028067128470764related:7GzejAtXWGsJ. 11227840 10.1353/dem.2001.0003

[32] Rutstein SO, Johnson K. The DHS Wealth Index. Calverton, Maryland: ORC Macro, 2004 8 01. Report No.

[33] Tobler WR. A Computer Movie Simulating Urban Growth in the Detroit Region. Economic Geography. 1970;46:234–40. 15192663253141002697related:yZmZC7wu19IJ.

[34] Aneselin L, Syabri I, Kho Y. GeoDa: An Introduction to Spatial Data Analysis. Geographic Analysis. 2006;38(1):5–22.

[35] Krivoruchko K. Empirical Bayesian Kriging—Implemented in ArcGIS Geostatistical Analyst. ArcUser. 2012;(Fall edition):6–10.

[36] UNICEF, WHO. Estimates for the use of improved sanitation facilities. 2010.

[37] Andersson N, Mitchell S. Epidemiological geomatics in evaluation of mine risk education in Afghanistan: introducing population weighted raster maps. International Journal of Health Geographics. 2006. 10357779503358452567related:V6OAl5Q5vo8J. 10.1186/1476-072X-5-1 PMC1352365 16390549

[38] Bohra A, Andrianasolo H. Application of GIS in modeling of dengue risk based on sociocultural data: case of Jalore, Rajasthan, India. Dengue Bull. 2001;25:92–102. 3364734259829277195related:CwK2cPvvsS4J.

[39] English PB, Kharrazi M, Davies S, Scalf R, Waller L, Neutra R. Changes in the spatial pattern of low birth weight in a southern California county: the role of individual and neighborhood level factors. Social Science & Medicine. 2003;56(10):2073–88. . 12697198 10.1016/s0277-9536(02)00202-2

[40] Gent DH, Schwartz HF, Khosla R. Distribution and Incidence of Iris yellow spot virus in Colorado and Its Relation to Onion Plant Population and Yield. Plant Disease. 2004;88(5):446–52. 6550463168897289468related:_Axn2Ivr51oJ. 10.1094/PDIS.2004.88.5.446 30812646

