# Correction: High-Resolution Spatial Distribution and Estimation of Access to Improved Sanitation in Kenya

**DOI:** 10.1371/journal.pone.0162580

**Published:** 2016-09-01

**Authors:** Peng Jia, John D. Anderson, Michael Leitner, Richard Rheingans

The affiliation for the third author is incorrect. Michael Leitner is not affiliated with #5 Department of Geoinformatics—Z_GIS, University of Salzburg, Salzburg, Austria

## References

[1] Jia P, Anderson JD, Leitner M, Rheingans R (2016) High-Resolution Spatial Distribution and Estimation of Access to Improved Sanitation in Kenya. PLoS ONE 11(7): e0158490 doi: 10.1371/journal.pone.0158490 27404271 10.1371/journal.pone.0158490 PMC4942115

