# Correction: High-Resolution Spatial Distribution and Estimation of Access to Improved Sanitation in Kenya

**DOI:** 10.1371/journal.pone.0165685

**Published:** 2016-10-24

**Authors:** Peng Jia, John D. Anderson, Michael Leitner, Richard Rheingans

There are errors in the Funding section. The correct funding information is as follows: "The research was funded by UK aid from the Department for International Development (DfID), as part of the SHARE (www.SHAREresearch.org) research programme.

## References

[1] Jia P, Anderson JD, Leitner M, Rheingans R (2016) High-Resolution Spatial Distribution and Estimation of Access to Improved Sanitation in Kenya. PLoS ONE 11(7): e0158490 doi: 10.1371/journal.pone.0158490 27404271 10.1371/journal.pone.0158490 PMC4942115

